# Piezo1 channels enhance anabolic signaling activation induced by electrical stimulation of cultured myotubes

**DOI:** 10.1002/2211-5463.70008

**Published:** 2025-02-17

**Authors:** Natalia A. Vilchinskaya, Ksenia V. Sergeeva, Boris S. Shenkman, Timur M. Mirzoev

**Affiliations:** ^1^ Myology Laboratory Institute of Biomedical Problems of the Russian Academy of Sciences Moscow Russia

**Keywords:** C2C12 myotubes, electrical pulse stimulation, gadolinium, Piezo1, protein synthesis, Yoda1

## Abstract

Mechanically activated (MA) Piezo1 channels play an important role in both normal physiology and pathological dysfunction in multiple tissues and organs. In skeletal muscle cells, Piezo1 channels are involved in the regulation of postnatal myogenesis and muscle regeneration after injury. To further understand the role of MA Piezo1 channels as potential critical sensors of mechanical perturbations during muscle contractions, we studied the possible contribution of MA Piezo1 channels to enhanced protein synthetic response of C2C12 myotubes to mechanical simulation. C2C12 myotubes were subjected to mechanical stimulation by electrical pulse stimulation (EPS) alone or EPS in combination with Yoda1, Gadolinium or Yoda1 + Gadolinium. EPS alone elicited an increase in anabolic signaling and protein synthesis (PS). Incubation with Yoda1 during EPS enhanced anabolic signaling and PS compared to EPS alone. Gadolinium or Yoda1 + gadolinium during EPS abolished or diminished the Yoda1 + EPS‐induced effects on anabolic signaling and PS. Our work demonstrates that chemical activation of Piezo1 channels during mechanical stimulation contributes to enhanced protein anabolism in C2C12 myotubes.

## Graphical abstract

By using an electrical pulse stimulation (EPS)‐based *in vitro* exercise model and chemical activation/inhibition of mechanosensitive Piezo1 channels, we explored changes in protein synthetic response in cultured C2C12 myotubes. Our data showed that application of Piezo1 activator (Yoda1) during EPS significantly enhances the rate of protein synthesis in C2C12 myotubes, possibly via mTORC1 pathway and inhibitory Ser9 phosphorylation of GSK‐3β.

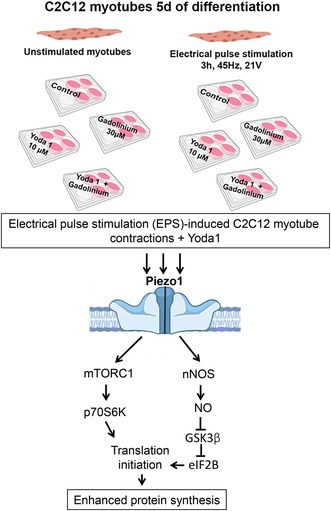

**Abbreviations**
AKT: protein kinase B
cGMP: cyclic guanosine monophosphate
eEF2: eukaryotic elongation factor 2
eEF2K: eukaryotic elongation factor 2 kinase
eIF2B: eukaryotic initiation factor 2B
EPS: electrical pulse stimulation
GC: guanylate cyclase
GSK‐3β: glycogen synthase kinase 3β
MA: mechanically activated
mTORC1: mechanistic target of rapamycin, complex 1
nNOS: neuronal NO synthase
NO: nitric oxide
p70S6K: ribosomal protein S6 kinase
PKG: protein kinase G
PS: protein synthesis
SUnSET: surface sensing of translation technique

Muscle cells/fibers are permanently exposed to mechanical forces during contractions/physical exercise which is essential for the maintenance and promotion of good health and quality of life [1, 2]. Since skeletal muscles experience and exert mechanical forces, each muscle fiber possesses special mechanosensory structures that are able to sense mechanical stress and convert it into a variety of biochemical pathways (mechanotransduction) that regulate different biological processes including protein synthesis and cell growth [3]. It has been recently uncovered that mechanically activated (MA) Piezo channels are critically important molecules in both normal physiology and pathological dysfunction in multiple tissues and organs [4, 5, 6]. As for skeletal muscle cells, it has been demonstrated that Piezo1 channels are involved in the regulation of such vital processes as postnatal myogenesis [7, 8] and muscle regeneration after injury [9, 10]. We hypothesized that MA Piezo1 channels may serve as critical sensors of mechanical perturbations during muscle contractions and are able to contribute to the regulation of exercise‐induced anabolic processes in cultured myotubes.

In order to clarify various physiological mechanisms involved in exercise‐induced adaptations, *in vitro* approaches using cultured muscle cells have been developed in the past 10–15 years [11, 12, 13, 14]. In particular, an electrical pulse stimulation (EPS)‐based *in vitro* exercise model has been proposed to mimic *in vivo* muscle responses to different types of exercise [11, 14, 15, 16, 17]. Such *in vitro* exercise model offers researchers a wide range of stimulation protocols eliciting contraction of cultured myotubes by means of changing EPS variables (voltage, frequency, and duration of pulses). Thus, the use of this model allows us to gain insight into molecular mechanisms regulating various physiological/metabolic processes induced by exercise/muscle contraction including protein synthesis.

It is well established that mechanical load in the form of resistance exercise results in increased rates of muscle protein synthesis and subsequent muscle growth [18]. Enhanced protein synthesis due to mechanical stress is known to be associated with the activation intracellular signaling cascades implicated in the regulation of mRNA translation initiation and elongation [19]. In the present study, we assessed the activity of several components of the pathways involved in the regulation of mRNA translation including the mTORC1/p70S6K, NO/GSK‐3β/eIF2B, and eEF2K/eEF2 pathways. Mechanistic target of rapamycin, complex 1 (mTORC1) is a key signaling protein required for the stimulation of muscle protein synthesis at the level of translation initiation [20, 21]. It is important to note that phosphorylation of a key downstream target of mTORC1, 70‐kDa ribosomal protein S6 kinase (p70S6K), correlates with an increase in skeletal muscle mass after resistance exercise [22].

Eukaryotic elongation factor 2 (eEF2) is known to be a key regulator of polypeptide chain elongation that catalyzes translocation of the ribosome along mRNA [23]. Phosphorylation of eEF2 at Thr56 by eukaryotic elongation factor 2 kinase (eEF2K) is known to result in the inhibition of translation elongation [23].

Glycogen synthase kinase 3β (GSK‐3β) has recently emerged as an endogenous negative regulator of protein anabolism in skeletal muscles [24]. The activity of this constitutively active kinase is usually determined by estimating the levels of its inhibitory Ser9 phosphorylation [24]. An important role in the regulation of GSK‐3 activity is attributed to nitric oxide (NO) since GSK‐3 can be deactivated via the classic NO/soluble guanylate cyclase (GC)/cyclic guanosine monophosphate (cGMP)/protein kinase G (PKG) signaling pathway [24]. Inactivation of GSK‐3β can result in dephosphorylation of eukaryotic initiation factor 2B (eIF2B) thereby promoting the initiation of mRNA translation [7].

Previous studies suggest that some unidentified mechanically gated channels can mediate mechanotransduction for muscle protein synthesis in response to mechanical load [8, 9, 10]. Although it has long been established that muscle contractions can induce an increase in protein anabolism, the potential contribution of mechanically activated Piezo1 channels to the regulation of contraction‐induced anabolic processes in skeletal muscles remains unclear. Therefore, using Yoda1 (Piezo1 activator) and gadolinium (an inhibitor of MA channels), the aim of the study was to assess possible contribution of Piezo1 channels to enhanced protein synthetic response in C2C12 myotubes after mechanical simulation in the form of EPS‐based *in vitro* exercise.

## Materials and methods

### Cell culture

Murine C2C12 skeletal myoblasts obtained from AddexBio Collection cell line P0028001 (AddexBio, San Diego, CA, USA) were cultured in growth medium (DMEM, 20% FBS, 1% Penicillin–Streptomycin–Amphotericin B, 1% L‐glutamine) (Gibco, ThermoFisher Scientific, Waltham, MA, USA) until 80% confluency was reached. Next, myogenic differentiation of C2C12 was induced by replacing the growth medium with differentiation medium consisting of DMEM (Gibco) supplemented with 1% L‐glutamine (Invitrogen, ThermoFisher Scientific, Waltham, MA, USA), 1% Penicillin–Streptomycin–Amphotericin B and 2% horse serum (Gibco). Dishes were kept at 37 °C in a humidified atmosphere of 5% CO_2_. The culture medium was renewed every 24 h.

### Electrical pulse stimulation and chemical treatments

On day 5 of differentiation, EPS using a C‐Pace EM multi‐channel stimulator (IonOptix, Westwood, MA, USA) was applied for 3 h, with a protocol consisting of 2 ms pulses at 21 V, with a frequency of 45 Hz. After completion of EPS, a recovery period of 4 h was applied. The EPS protocol was aimed at inducing a significant anabolic response (an increase in both p70S6K phosphorylation and protein synthesis) and based upon our pilot EPS studies and available literature [16]. The EPS protocol used in the current study did not result in any visible cell detachment, and estimation of lactate dehydrogenase (LDH) release in culture media using a colorimetric cytotoxicity assay (CytoTox 96^®^ Non‐Radioactive Cytotoxicity Assay; Promega, Madison, WI, USA) showed that LDH was unchanged in medium from stimulated vs. unstimulated cells. Thirty minutes before the start of EPS, fresh differentiation medium or medium containing Yoda1 (10 μm) alone (CAS 448947‐81‐7, Tocris Bioscience, Bristol, UK), Gd^3+^ (30 μm) alone (# sc‐224 004, Santa Cruz Biotechnology, Santa Cruz, CA, USA), or Yoda1 combined with Gd^3+^ were added to C2C12 myotubes. Yoda1 and Gd^3+^ were dissolved in 0.1% dimethylsulfoxide (DMSO). Electrically stimulated but untreated cells were incubated with vehicle (0.1% DMSO) alone. The concentrations for Yoda1 and Gd^3+^ and treatment duration used in the study were based on previously published work [25, 26] and testing that preceded the experiment. Three and a half hours after the completion of EPS, the medium was replaced with fresh differentiation medium containing 1 μm puromycin in order to determine the rate of protein synthesis (see below). Myotubes were then rinsed with PBS and frozen at – 80 °C until further analysis.

### Measurements of protein synthesis

Surface sensing of translation (SUnSET) is a nonradioactive technique that allows to measure protein synthesis in skeletal muscle cells/fibers both *in vitro* and *in vivo* [27]. For measurements of protein synthesis in the current study, C2C12 myotubes were incubated with 1 μm puromycin (BML‐GR312, Enzo Life Sciences, Long Island, New York, USA) for 30 min before collection. The SUnSET technique involves the use of the antibiotic puromycin (a structural analogue of tyrosyl‐tRNA), and anti‐puromycin antibodies to detect the amount of puromycin incorporation into nascent peptide chains. In order to visualize and quantify, the rates of protein synthesis the SUnSET technique uses standard western blotting analysis as described below.

### Western blot analysis

Skeletal myotubes were washed with PBS, lysed in RIPA buffer (for 30 min at 4 °C), and then centrifugated at 12 000 ***g*** for 10 min at 4 °C. Protein concentrations were determined by Bradford protein assay (#5000205, Bio‐Rad, Bio‐Rad Laboratories, CA, USA). Western blot analysis was performed as described in our previous studies [9, 28]. Primary antibodies used in the study were as follows: p‐AKT (Ser473) (1 : 2000, cat. #9271, Cell Signaling, Danvers, MA, USA), AKT (1 : 2000, cat. #9272, Cell Signaling), p‐GSK3beta (Ser9) (1 : 1000, cat. #9322 Cell Signaling), GSK‐3beta (1 : 2000, cat. #12456, Cell Signaling), p‐p70S6K (Thr389) (1 : 500, cat. # AF3228, Affinity Biosciences, Cincinnati, OH, USA), p70S6K (1 : 3000, cat. #9202, Cell Signaling), p‐eEF2 (Thr56) (1 : 500, cat. #2331, Cell Signaling), eEF2 (1 : 2000, cat. #2332, Cell Signaling), p‐nNOS (Ser1417) (1 : 500, #AF8231, Affinity Biosciences), nNOS (1 : 1000, #610309, BD Biosciences, Franklin Lakes, NJ, USA), GAPDH (1 : 3000, cat. # AF7021, Affinity Biosciences), and puromycin (clone 12D10) (1 : 2000, cat. #MABE343, Merck, Burlington, MA, USA). Horseradish peroxidase‐conjugated antibodies to rabbit immunoglobulins (1 : 60 000, cat. # 111‐035‐003, Jackson Immuno Research, Cambridge, UK) were used as secondary antibodies. Following image capture of phosphorylated proteins, membranes were stripped of the phospho‐specific antibodies using RestoreTM Western Blot Stripping Buffer (cat. # 21059, Thermo Fisher, ThermoFisher Scientific, Waltham, MA, USA). The membranes were then reprobed with primary antibodies for each respective total protein. Protein expression of phosphorylated proteins was normalized to corresponding total proteins. For detection of the rate of protein synthesis (SUnSET technique), the measurements of the chemiluminescent signals were performed by determining the density of each whole lane with the entire molecular weight range of puromycin‐labeled peptides. The Ponceau S staining of the total protein was used for normalization in the quantification of the content of puromycin‐labeled proteins (the rate of protein synthesis). The protein bands/lanes were quantified using C‐DiGit Blot Scanner and Image Studio Digits software (LI‐COR Biotechnology, Lincoln, NE, USA).

### Statistical analysis

Statistical analysis was performed using graphpad prism 8.0 software (La Jolla, CA, USA). qRT‐PCR and western blot data are shown as mean ± SEM. Two‐way ANOVA with *post hoc* Tukey test was used to determine the significant differences between group means. Statistical significance was accepted at *P* < 0.05.

## Results

**Fig. 1:**
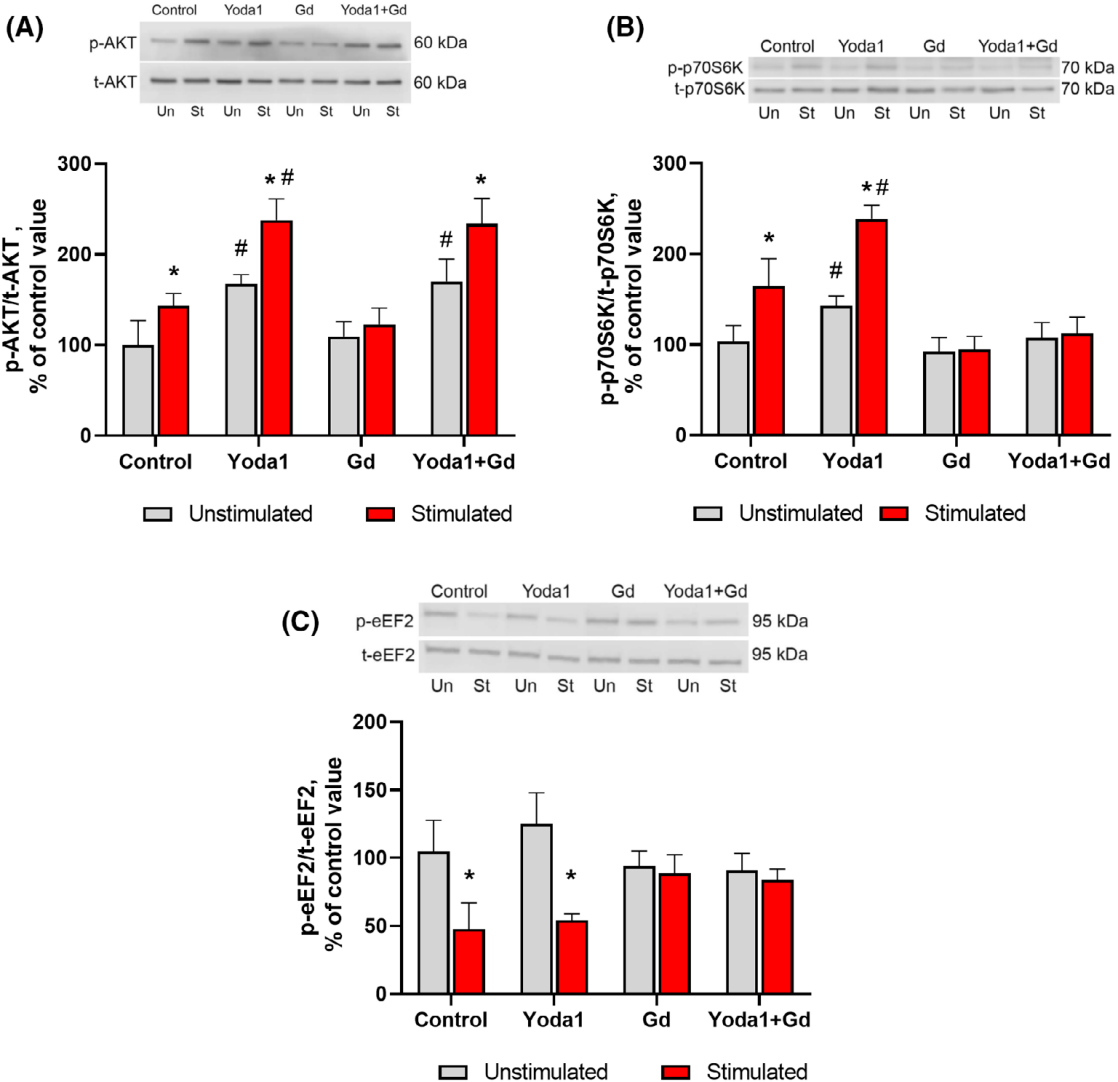
Phosphorylation status of signaling molecules involved in the regulation of protein synthesis in response to EPS of myotubes and concomitant treatment with Yoda1 and/or Gd3+. (A) Phosphorylated (Thr 473) to total ratio of AKT. (B) Phosphorylated (Thr389) to total ratio of p70S6K. (C) Phosphorylated (Thr56) to total ratio of eEF2. Gray bars: cells were not subjected to EPS (unstimulated); red bars: cells were subjected to EPS (stimulated). Data are expressed as a percentage of the mean value obtained in the control (unstimulated) cells and are reported as the mean ± SEM., *n* = 5–6/group. Two‐way ANOVA with *post hoc* Tukey test was used to determine significant differences between the groups. *—significantly different from the unstimulated cells in each group, *P* < 0.05, #—significantly different from the unstimulated or stimulated cells of the control group, *P* < 0.05.

**Fig. 2:**
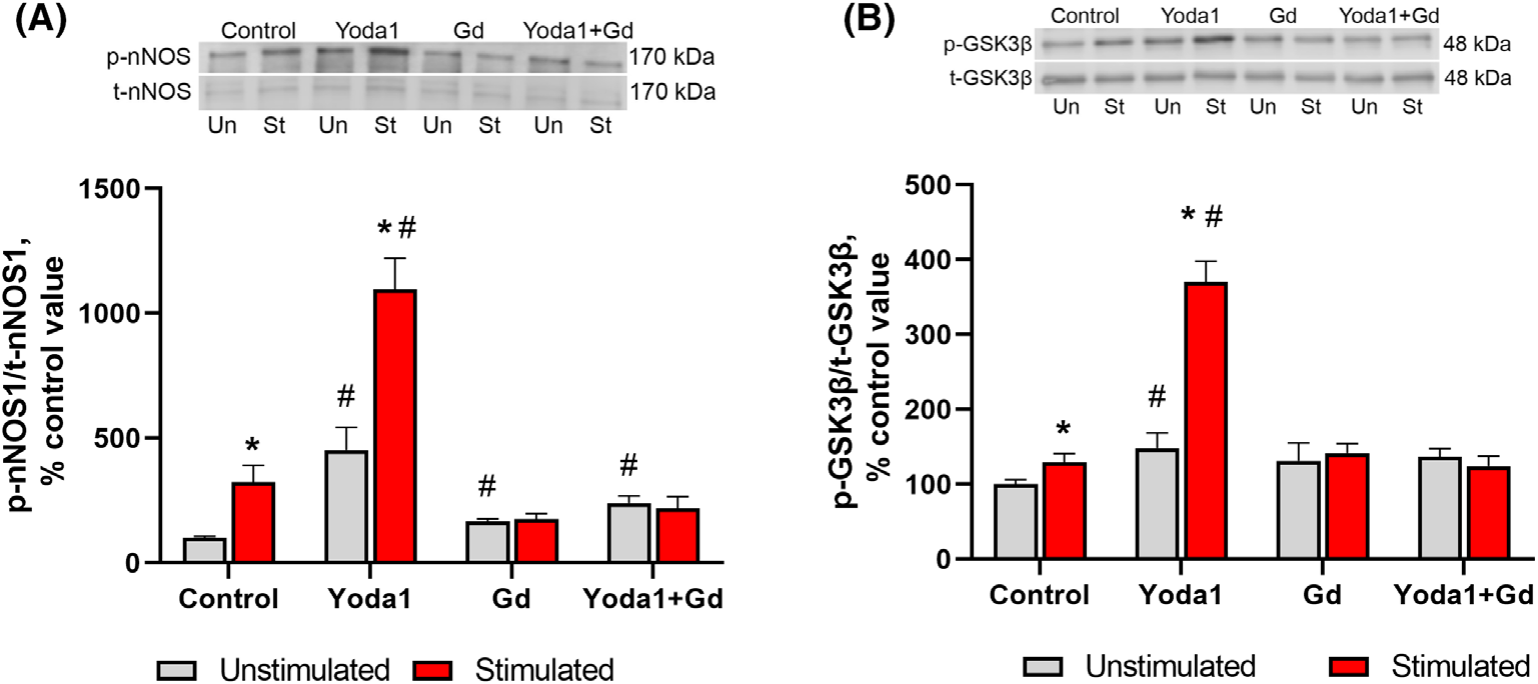
Phosphorylation status of nNOS (Ser1417) (A) and GSK‐3β (Ser9) (B) in response to EPS of myotubes and concomitant treatment with Yoda1 and/or Gd3+. Gray bars: cells were not subjected to EPS (unstimulated); red bars: cells were subjected to EPS (stimulated). Data are expressed as a percentage of the mean value obtained in the control (unstimulated) cells and are reported as the mean ± SEM., *n* = 5–6/group. Two‐way ANOVA with *post hoc* Tukey test was used to determine significant differences between the groups. *—significantly different from the unstimulated cells in each group, *P* < 0.05, #—significantly different from the unstimulated or stimulated cells of the control group, *P* < 0.05.

**Fig. 3:**
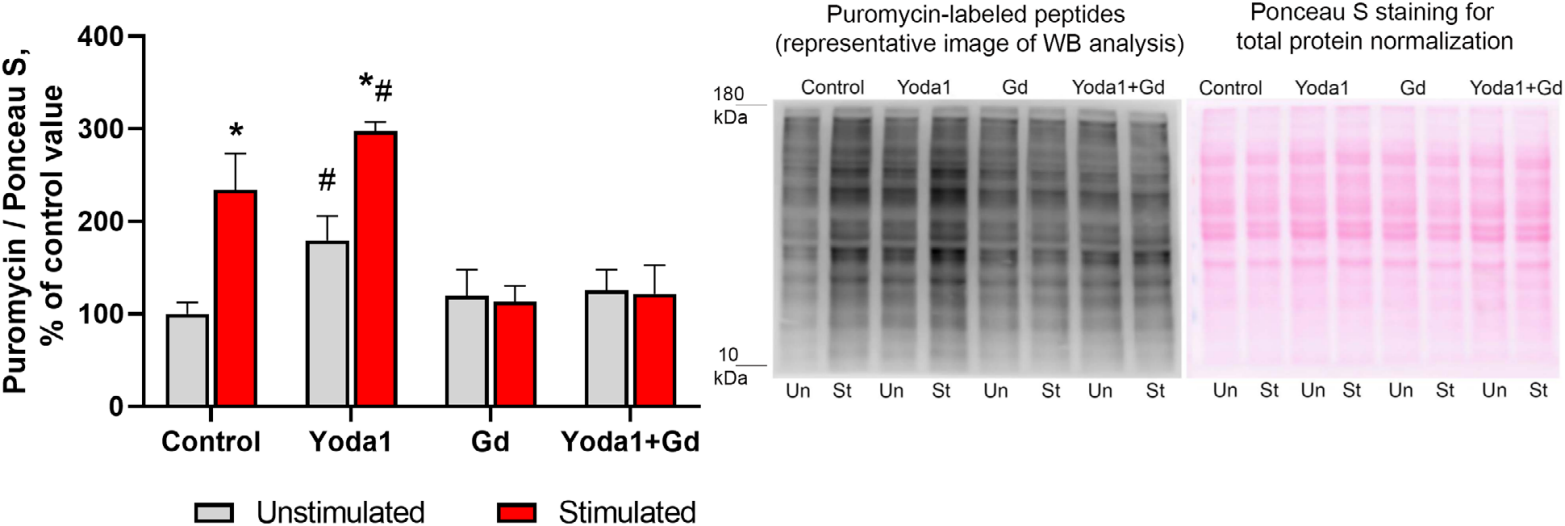
Changes in the rates of protein synthesis in response to EPS of myotubes and concomitant treatment with Yoda1 and/or Gd3+. Gray bars: cells were not subjected to EPS (unstimulated); red bars: cells were subjected to EPS (stimulated). Data are expressed as a percentage of the mean value obtained in the control (unstimulated) cells and are reported as the mean ± SEM., *n* = 5‐6/group. Two‐way ANOVA with *post hoc* Tukey test was used to determine significant differences between the groups. *—significantly different from the unstimulated cells in each group, *P* < 0.05, #—significantly different from the unstimulated or stimulated cells of the control group, *P* < 0.05.

As expected, EPS of C2C12 myotubes resulted in an activation of the AKT/mTORC1/p70S6K signaling pathway which plays a key role in the regulation of mRNA translation initiation. Indeed, phosphorylation of AKT (Ser473) and p70S6K (Thr389) significantly increased by 43% and 80% (*P* < 0.05), respectively, 4 h after completion of EPS compared to the nonstimulated (control) myotubes (Fig. 1A,B). Moreover, a significant decrease in eEF2 (Thr56) phosphorylation (−62%, *P* < 0.05) after EPS was indicative of enhanced mRNA translation elongation (Fig. 1C). In order to assess a possible contribution of the NO/GSK‐3β pathway to protein synthesis, we determined phosphorylation levels of neuronal NO synthase (nNOS) and GSK‐3β (Ser9) phosphorylation. EPS induced a robust increase in nNOS (Ser1417) phosphorylation by 223% (*P* < 0.05) and concomitant increase in phosphorylation levels of GSK‐3β (Ser9) (+31%, *P* < 0.05) (Fig. 2), indicative of nNOS activation and GSK‐3β inhibition. These signaling events were correlated with increased rates of protein synthesis (+134%, *P* < 0.05) following the exposure of myotubes to EPS as measured by the SUnSET technique (Fig. 3).

Treatment of myotubes with Yoda1 during EPS resulted in a significant acceleration of phosphorylation of AKT, p70S6K (Fig. 1), nNOS and GSK‐3β (Fig. 2) compared to EPS without Yoda1 treatment. Moreover, application of Yoda1 during EPS also elicited enhanced rates of protein synthesis (+63%, *P* < 0.05) (Fig. 3), while not affecting eEF2 phosphorylation (Fig. 1C), in comparison with the untreated EPS myotubes.

Exposure of myotubes to Gd^3+^ alone as well as combined Yoda1 + Gd^3+^ treatment completely abolished EPS‐induced and EPS + Yoda1‐induced changes in both phosphorylation of the studied signaling molecules (except for AKT phosphorylation in the Yoda1 + Gd^3+^ cells) and the rates of protein synthesis.

## Discussion

In this study, we employed an *in vitro* muscle contraction model to induce protein synthetic response and assess possible contribution of MA Piezo1 channels to the regulation of contraction‐related protein anabolism in C2C12 myotubes. Contraction‐induced activation of the mTORC1 signaling pathway, translation elongation and protein synthesis in myotubes, observed in the current study, is in line with previous reports that demonstrated a significant enhancement of protein anabolism in response to different types of mechanical stimulation [19]. How can mechanical signals induced by muscle contractions be transduced to biochemical events activating protein synthesis? Literature suggests that mechanical deformation of muscle cells/fibers may activate protein synthesis via (a) yet unidentified mechanosensory proteins acting on the zeta isoform of diacylglycerol kinase, which leads to the conversion of diacylglycerol into phosphatidic acid and subsequent mTORC1 activation [29]; (b) unidentified mechano‐sensing kinase that is able to induce tuberous sclerosis complex‐2 phosphorylation and translocation away from the lysosome leading to mTORC1 activation [30]. We hypothesized that MA Piezo1 channels may serve as potential regulators of muscle anabolism in response to mechanical stimuli. MA channels have gained our attention since previous studies showed that eccentric contractions‐induced activation of mTORC1/protein synthesis in skeletal muscles can be attenuated by gadolinium treatment [9, 10]. Moreover, an increase in muscle protein synthesis in rat soleus muscle associated with acute recovery following mechanical unloading is attenuated with inhibition of stretch‐activated channels [8]. In addition, a recent study from our lab revealed that incubation of isolated soleus muscle with Gd^3+^ is able to prevent passive stretch‐related activation of the mTORC1 signaling [31]. The inhibitory effect of Gd^3+^ on the EPS‐induced activation of anabolic parameters, observed in the present study, supports the above‐mentioned whole‐muscle data.

According to Fig. 1A, treatment of myotubes with Gd^3+^ did not abolish Yoda1‐induced or Yoda1 + EPS‐induced increases in AKT (Ser473) phosphorylation. One possible reason why Gd^3+^ did not block AKT activity is that Yoda1‐induced phosphorylation of AKT (Ser473) may not be directly related to Piezo1 channels. This hypothesis is supported by the fact that pretreatment of endothelial cells with GsMTx4, an inhibitor of MA channels including Piezo1, does not prevent Yoda1‐induced AKT (Ser473) phosphorylation. This suggests that Yoda1 activates AKT in endothelial cells through mechanisms that are not fully dependent on Piezo1 activation [32]. Another hypothesis is that the concentration of Gd^3+^ and/or duration of its use in our research were not enough to reduce Yoda1‐induced activation of Piezo1 and, consequently, to weaken the phosphorylation of AKT.

**Fig. 4:**
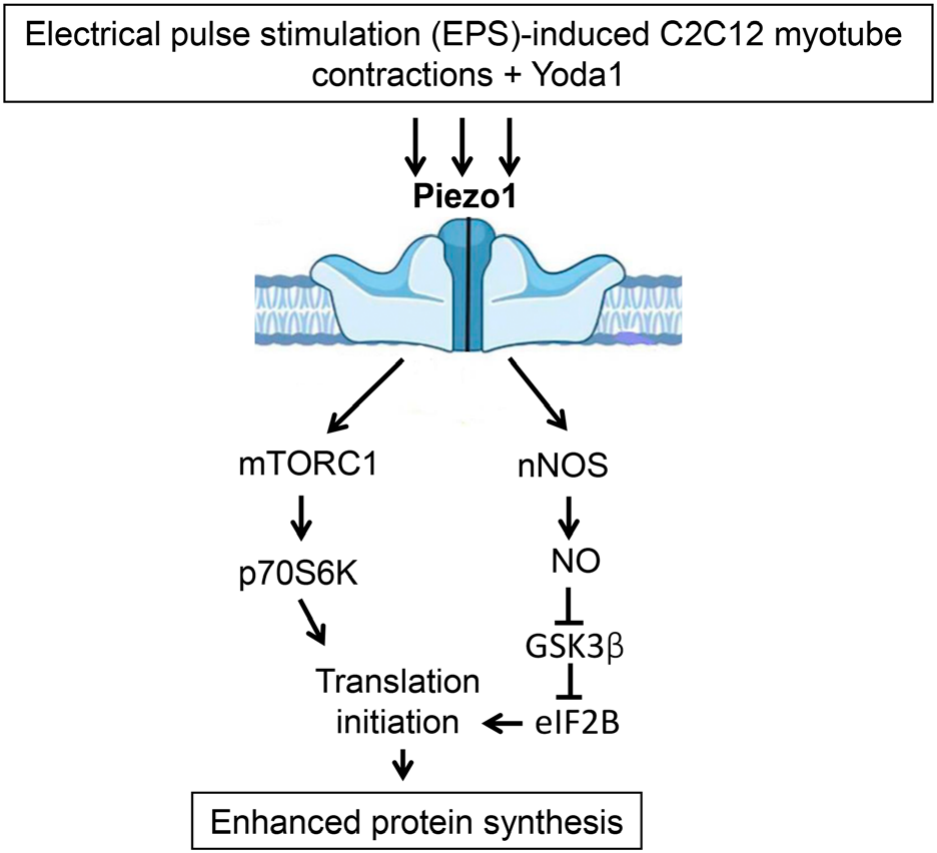
Signaling pathways involved in Piezo1‐related enhancement of protein synthesis in cultured C2C12 myotubes in response to EPS. Activation of Piezo1 channels with Yoda1 during EPS significantly enhanced the activity of mTORC1/p70S6K pathway and inhibitory Ser9 phosphorylation of GSK‐3β thereby influencing the rate of protein synthesis in cultures myotubes. Enhanced GSK‐3β Ser9 phosphorylation (i.e., inhibition) could be associated with increased NO production induced by nNOS activation.

An important finding of our study is that Yoda1 treatment was able to enhance an anabolic response in myotubes after a period of mechanical stimulation in the form of EPS. Since Piezo1 channels are permeable to calcium ions [33] and application of Yoda1 can induce a rise in intracellular calcium levels in muscle cells [25], an enhanced anabolic response could be related to changes in myoplasmic calcium concentration. Literature suggests that, first, a key anabolic molecule, p70S6K, requires an initial calcium‐dependent priming event in order to become fully activated [34, 35]. Second, it has been demonstrated that an increase in intracellular calcium levels in muscle cells/fibers can induce mTORC1 signaling activation [36, 37]. Moreover, Ca^2+^/calmodulin is also implicated in the activation of nNOS [38, 39] and thereby NO production. In the present study, Yoda1 treatment significantly enhanced nNOS activity in response to EPS, possibly due to a Piezo1‐related increase in the intracellular calcium levels. Interestingly, it has been previously demonstrated that mechanically activated channels are involved in fluid shear stress‐induced NO production by C2C12 cells [40]. This Yoda1‐related increase in NO in response to Yoda1 treatment could contribute to a significant increase in inhibitory GSK‐3β Ser9 phosphorylation via the NO/cGMP/PKG pathway [41]. As GSK‐3β may act as an endogenous suppressor of protein anabolism [24], its hyperphosphorylation (i.e., inactivation) could contribute to accelerated rates of protein synthesis in response to EPS + Yoda1 treatment. Thus, the results of the present study suggest that Yoda1‐induced (i.e., Piezo1‐related) acceleration of protein synthesis in cultured myotubes in response to EPS may be associated with enhanced activity of the mTORC1/p70S6K and nNOS/GSK‐3β signaling pathways (Fig. 4).

Several recent studies have shown that Piezo1 channels may crosstalk with a mechanosensitive transcriptional co‐activator Yes‐associated protein (YAP) in different types of cells [42, 43, 44, 45]. Since YAP overexpression was shown to be involved in increased muscle protein synthesis [46] and skeletal muscle hypertrophy [46, 47], it is not excluded that an enhanced anabolic response in the EPS + Yoda1 myotubes could be associated with the activation of Piezo1/YAP signaling pathway.

In conclusion, the present work shows that chemical activation of Piezo1 channels significantly enhances contraction‐induced activation of anabolic signaling and protein synthesis in C2C12 myotubes. Thus, pharmacological activation of Piezo1 channels during muscle contractions may represent a new effective tool to promote exercise‐induced protein synthesis in skeletal muscles.

## Conflict of interest

The authors declare no conflict of interest.

## Author contributions

NAV, TMM, and BSS: conceptualization; NAV, TMM, and BSS: methodology; NAV and KVS: formal analysis and investigation; TMM: writing—original draft preparation; NAV: writing—review and editing; TMM: funding acquisition. All authors have read and approved the final manuscript.

## Data Availability

All data concerning the study are available on reasonable request from the corresponding author.

## References

[1] Wolfe RR (2006) The underappreciated role of muscle in health and disease. Am J Clin Nutr 84, 475–482. 16960159 10.1093/ajcn/84.3.475

[2] Vina J , Rodriguez‐Manas L , Salvador‐Pascual A , Tarazona‐Santabalbina FJ and Gomez‐Cabrera MC (2016) Exercise: the lifelong supplement for healthy ageing and slowing down the onset of frailty. J Physiol 594, 1989–1999. 26872560 10.1113/JP270536 PMC4933124

[3] Olsen LA , Nicoll JX and Fry AC (2019) The skeletal muscle fiber: a mechanically sensitive cell. Eur J Appl Physiol 119, 333–349. 30612167 10.1007/s00421-018-04061-x

[4] Syeda R , Florendo MN , Cox CD , Kefauver JM , Santos JS , Martinac B and Patapoutian A (2016) Piezo1 channels are inherently mechanosensitive. Cell Rep 17, 1739–1746. 27829145 10.1016/j.celrep.2016.10.033 PMC5129625

[5] Coste B , Xiao B , Santos JS , Syeda R , Grandl J , Spencer KS , Kim SE , Schmidt M , Mathur J , Dubin AE *et al*. (2012) Piezo proteins are pore‐forming subunits of mechanically activated channels. Nature 483, 176–181. 22343900 10.1038/nature10812 PMC3297710

[6] Fang XZ , Zhou T , Xu JQ , Wang YX , Sun MM , He YJ , Pan SW , Xiong W , Peng ZK , Gao XH *et al*. (2021) Structure, kinetic properties and biological function of mechanosensitive piezo channels. Cell Biosci 11, 13. 33422128 10.1186/s13578-020-00522-z PMC7796548

[7] Welsh GI , Miller CM , Loughlin AJ , Price NT and Proud CG (1998) Regulation of eukaryotic initiation factor eIF2B: glycogen synthase kinase‐3 phosphorylates a conserved serine which undergoes dephosphorylation in response to insulin. FEBS Lett 421, 125–130. 9468292 10.1016/s0014-5793(97)01548-2

[8] Mirzoev TM , Tyganov SA , Petrova IO and Shenkman BS (2019) Acute recovery from disuse atrophy: the role of stretch‐activated ion channels in the activation of anabolic signaling in skeletal muscle. Am J Physiol Endocrinol Metab 316, E86–E95. 30457911 10.1152/ajpendo.00261.2018

[9] Tyganov S , Mirzoev T and Shenkman B (2019) An anabolic signaling response of rat soleus muscle to eccentric contractions following Hindlimb unloading: a potential role of stretch‐activated ion channels. Int J Mol Sci 20, 1165. 30866432 10.3390/ijms20051165 PMC6429234

[10] Spangenburg EE and McBride TA (2006) Inhibition of stretch‐activated channels during eccentric muscle contraction attenuates p70S6K activation. J Appl Physiol 100, 129–135. 16179399 10.1152/japplphysiol.00619.2005

[11] Nedachi T , Fujita H and Kanzaki M (2008) Contractile C2C12 myotube model for studying exercise‐inducible responses in skeletal muscle. Am J Physiol Endocrinol Metab 295, E1191–E1204. 18780777 10.1152/ajpendo.90280.2008

[12] Lambernd S , Taube A , Schober A , Platzbecker B , Gorgens SW , Schlich R , Jeruschke K , Weiss J , Eckardt K and Eckel J (2012) Contractile activity of human skeletal muscle cells prevents insulin resistance by inhibiting pro‐inflammatory signalling pathways. Diabetologia 55, 1128–1139. 22282161 10.1007/s00125-012-2454-z

[13] Nikolic N , Bakke SS , Kase ET , Rudberg I , Flo Halle I , Rustan AC , Thoresen GH and Aas V (2012) Electrical pulse stimulation of cultured human skeletal muscle cells as an in vitro model of exercise. PLoS One 7, e33203. 22457744 10.1371/journal.pone.0033203 PMC3310863

[14] Tarum J , Folkesson M , Atherton PJ and Kadi F (2017) Electrical pulse stimulation: an in vitro exercise model for the induction of human skeletal muscle cell hypertrophy. A proof‐of‐concept study. Exp Physiol 102, 1405–1413. 28861930 10.1113/EP086581

[15] Sato S , Nomura M , Yamana I , Uchiyama A , Furuichi Y , Manabe Y and Fujii NL (2019) A new in vitro muscle contraction model and its application for analysis of mTORC1 signaling in combination with contraction and beta‐hydroxy‐beta‐methylbutyrate administration. Biosci Biotechnol Biochem 83, 1851–1857. 31159662 10.1080/09168451.2019.1625261

[16] Nikolic N , Gorgens SW , Thoresen GH , Aas V , Eckel J and Eckardt K (2017) Electrical pulse stimulation of cultured skeletal muscle cells as a model for in vitro exercise – possibilities and limitations. Acta Physiol 220, 310–331. 10.1111/apha.12830 27863008

[17] Manabe Y , Miyatake S , Takagi M , Nakamura M , Okeda A , Nakano T , Hirshman MF , Goodyear LJ and Fujii NL (2012) Characterization of an acute muscle contraction model using cultured C2C12 myotubes. PLoS One 7, e52592. 23300713 10.1371/journal.pone.0052592 PMC3534077

[18] Damas F , Phillips S , Vechin FC and Ugrinowitsch C (2015) A review of resistance training‐induced changes in skeletal muscle protein synthesis and their contribution to hypertrophy. Sports Med 45, 801–807. 25739559 10.1007/s40279-015-0320-0

[19] Goodman CA (2019) Role of mTORC1 in mechanically induced increases in translation and skeletal muscle mass. J Appl Physiol 127, 581–590. 30676865 10.1152/japplphysiol.01011.2018

[20] Drummond MJ , Fry CS , Glynn EL , Dreyer HC , Dhanani S , Timmerman KL , Volpi E and Rasmussen BB (2009) Rapamycin administration in humans blocks the contraction‐induced increase in skeletal muscle protein synthesis. J Physiol 587, 1535–1546. 19188252 10.1113/jphysiol.2008.163816 PMC2678224

[21] Dickinson JM , Fry CS , Drummond MJ , Gundermann DM , Walker DK , Glynn EL , Timmerman KL , Dhanani S , Volpi E and Rasmussen BB (2011) Mammalian target of rapamycin complex 1 activation is required for the stimulation of human skeletal muscle protein synthesis by essential amino acids. J Nutr 141, 856–862. 21430254 10.3945/jn.111.139485 PMC3077888

[22] Baar K and Esser K (1999) Phosphorylation of p70(S6k) correlates with increased skeletal muscle mass following resistance exercise. Am J Phys 276, C120–C127. 10.1152/ajpcell.1999.276.1.C120 9886927

[23] Browne GJ and Proud CG (2002) Regulation of peptide‐chain elongation in mammalian cells. Eur J Biochem 269, 5360–5368. 12423334 10.1046/j.1432-1033.2002.03290.x

[24] Mirzoev TM , Sharlo KA and Shenkman BS (2021) The role of GSK‐3β in the regulation of protein turnover, myosin phenotype, and oxidative capacity in skeletal muscle under disuse conditions. Int J Mol Sci 22, 5081. 34064895 10.3390/ijms22105081 PMC8151958

[25] Bosutti A , Giniatullin A , Odnoshivkina Y , Giudice L , Malm T , Sciancalepore M , Giniatullin R , D'Andrea P , Lorenzon P and Bernareggi A (2021) Time window effect of Yoda1‐evoked Piezo1 channel activity during mouse skeletal muscle differentiation. Acta Physiol 233, e13702. 10.1111/apha.13702 PMC9286833 34097801

[26] Sciancalepore M , Massaria G , Tramer F , Zacchi P , Lorenzon P and Bernareggi A (2022) A preliminary study on the role of Piezo1 channels in myokine release from cultured mouse myotubes. Biochem Biophys Res Commun 623, 148–153. 35914353 10.1016/j.bbrc.2022.07.059

[27] Goodman CA , Mabrey DM , Frey JW , Miu MH , Schmidt EK , Pierre P and Hornberger TA (2011) Novel insights into the regulation of skeletal muscle protein synthesis as revealed by a new nonradioactive in vivo technique. FASEB J 25, 1028–1039. 21148113 10.1096/fj.10-168799 PMC3042844

[28] Mirzoev TM , Tyganov SA and Shenkman BS (2017) Akt‐dependent and Akt‐independent pathways are involved in protein synthesis activation during reloading of disused soleus muscle. Muscle Nerve 55, 393–399. 27367189 10.1002/mus.25235

[29] You JS , Lincoln HC , Kim CR , Frey JW , Goodman CA , Zhong XP and Hornberger TA (2014) The role of diacylglycerol kinase zeta and phosphatidic acid in the mechanical activation of mammalian target of rapamycin (mTOR) signaling and skeletal muscle hypertrophy. J Biol Chem 289, 1551–1563. 24302719 10.1074/jbc.M113.531392 PMC3894336

[30] Jacobs BL , You JS , Frey JW , Goodman CA , Gundermann DM and Hornberger TA (2013) Eccentric contractions increase the phosphorylation of tuberous sclerosis complex‐2 (TSC2) and alter the targeting of TSC2 and the mechanistic target of rapamycin to the lysosome. J Physiol 591, 4611–4620. 23732640 10.1113/jphysiol.2013.256339 PMC3784202

[31] Sergeeva KV , Tyganov SA , Kalashnikov VE , Shenkman BS and Mirzoev TM (2023) Analysis of the role of Piezo1 channels in Mechano‐anabolic coupling in rat soleus muscle. Biol Membrany 40, 362–369.

[32] Dela Paz NG and Frangos JA (2018) Yoda1‐induced phosphorylation of Akt and ERK1/2 does not require Piezo1 activation. Biochem Biophys Res Commun 497, 220–225. 29428723 10.1016/j.bbrc.2018.02.058 PMC5835220

[33] Coste B , Mathur J , Schmidt M , Earley TJ , Ranade S , Petrus MJ , Dubin AE and Patapoutian A (2010) Piezo1 and Piezo2 are essential components of distinct mechanically activated cation channels. Science 330, 55–60. 20813920 10.1126/science.1193270 PMC3062430

[34] Conus NM , Hemmings BA and Pearson RB (1998) Differential regulation by calcium reveals distinct signaling requirements for the activation of Akt and p70S6k. J Biol Chem 273, 4776–4782. 9468542 10.1074/jbc.273.8.4776

[35] Hannan KM , Thomas G and Pearson RB (2003) Activation of S6K1 (p70 ribosomal protein S6 kinase 1) requires an initial calcium‐dependent priming event involving formation of a high‐molecular‐mass signalling complex. Biochem J 370, 469–477. 12429015 10.1042/BJ20021709 PMC1223178

[36] Ito N , Ruegg UT , Kudo A , Miyagoe‐Suzuki Y and Takeda S (2013) Activation of calcium signaling through Trpv1 by nNOS and peroxynitrite as a key trigger of skeletal muscle hypertrophy. Nat Med 19, 101–106. 23202294 10.1038/nm.3019

[37] Ito N , Ruegg UT and Takeda S (2018) ATP‐induced increase in intracellular calcium levels and subsequent activation of mTOR as regulators of skeletal muscle hypertrophy. Int J Mol Sci 19, 2804. 30231482 10.3390/ijms19092804 PMC6163678

[38] Du Bois P , Pablo Tortola C , Lodka D , Kny M , Schmidt F , Song K , Schmidt S , Bassel‐Duby R , Olson EN and Fielitz J (2015) Angiotensin II induces skeletal muscle atrophy by activating TFEB‐mediated MuRF1 expression. Circ Res 117, 424–436. 26137861 10.1161/CIRCRESAHA.114.305393 PMC4537692

[39] Forstermann U and Sessa WC (2012) Nitric oxide synthases: regulation and function. Eur Heart J 33, 829–837. 21890489 10.1093/eurheartj/ehr304 PMC3345541

[40] Juffer P , Bakker AD , Klein‐Nulend J and Jaspers RT (2014) Mechanical loading by fluid shear stress of myotube glycocalyx stimulates growth factor expression and nitric oxide production. Cell Biochem Biophys 69, 411–419. 24402674 10.1007/s12013-013-9812-4

[41] Drenning JA , Lira VA , Simmons CG , Soltow QA , Sellman JE and Criswell DS (2008) Nitric oxide facilitates NFAT‐dependent transcription in mouse myotubes. Am J Physiol Cell Physiol 294, C1088–C1095. 18272817 10.1152/ajpcell.00523.2007

[42] Zhong G , Su S , Li J , Zhao H , Hu D , Chen J , Li S , Lin Y , Wen L , Lin X *et al*. (2023) Activation of Piezo1 promotes osteogenic differentiation of aortic valve interstitial cell through YAP‐dependent glutaminolysis. Sci Adv 9, eadg0478. 37267365 10.1126/sciadv.adg0478 PMC10413650

[43] Mao J , Yang R , Yuan P , Wu F , Wei Y , Nie Y , Zhang C and Zhou X (2023) Different stimuli induce endothelial dysfunction and promote atherosclerosis through the Piezo1/YAP signaling axis. Arch Biochem Biophys 747, 109755. 37714252 10.1016/j.abb.2023.109755

[44] Ni K , Che B , Gu R , Wang C , Pan Y , Li J , Liu L , Luo M and Deng L (2024) Single‐cell hypertrophy promotes contractile function of cultured human airway smooth muscle cells via Piezo1 and YAP auto‐regulation. Cells 13, 1697. 39451215 10.3390/cells13201697 PMC11505810

[45] Lin CY , Sassi A , Wu Y , Seaman K , Tang W , Song X , Bienenstock R , Yokota H , Sun Y , Geng F *et al*. (2024) Mechanotransduction pathways regulating YAP nuclear translocation under Yoda1 and vibration in osteocytes. Bone 190, 117283. 39413946 10.1016/j.bone.2024.117283

[46] Watt KI , Turner BJ , Hagg A , Zhang X , Davey JR , Qian H , Beyer C , Winbanks CE , Harvey KF and Gregorevic P (2015) The hippo pathway effector YAP is a critical regulator of skeletal muscle fibre size. Nat Commun 6, 6048. 25581281 10.1038/ncomms7048

[47] Goodman CA , Dietz JM , Jacobs BL , McNally RM , You JS and Hornberger TA (2015) Yes‐associated protein is up‐regulated by mechanical overload and is sufficient to induce skeletal muscle hypertrophy. FEBS Lett 589, 1491–1497. 25959868 10.1016/j.febslet.2015.04.047 PMC4442043

